# Trophic Specialization Results in Genomic Reduction in Free-Living Marine *Idiomarina* Bacteria

**DOI:** 10.1128/mBio.02545-18

**Published:** 2019-01-15

**Authors:** Qi-Long Qin, Yi Li, Lin-Lin Sun, Zhi-Bin Wang, Shi Wang, Xiu-Lan Chen, Aharon Oren, Yu-Zhong Zhang

**Affiliations:** a State Key Laboratory of Microbial Technology, Marine Biotechnology Research Center, Shandong University, Qingdao, China; b College of Marine Life Sciences, Ocean University of China, Qingdao, China; c Laboratory for Marine Biology and Biotechnology, Qingdao National Laboratory for Marine Science and Technology, Qingdao, China; d Department of Plant and Environmental Sciences, The Alexander Silberman Institute of Life Sciences, Edmond J. Safra Campus, The Hebrew University of Jerusalem, Jerusalem, Israel; Oregon State University; RMIT University, Melbourne, Australia; The Chinese University of Hong Kong

**Keywords:** genome, *Idiomarina*, marine bacteria, trophic specialization

## Abstract

The streamlining hypothesis is generally used to explain the genomic reduction events related to the small genome size of free-living bacteria like marine bacteria SAR11. However, our current understanding of the correlation between bacterial genome size and environmental adaptation relies on too few species. It is still unclear whether there are other paths leading to genomic reduction in free-living bacteria. The genome size of marine free-living bacteria of the genus *Idiomarina* belonging to the order *Alteromonadales* (*Gammaproteobacteria*) is much smaller than the size of related genomes from bacteria in the same order. Comparative genomic and physiological analyses showed that the genomic reduction pattern in this genus is different from that of the classical SAR11 lineage. Genomic reduction reconstruction and substrate utilization profile showed that *Idiomarina* spp. lost a large number of genes related to carbohydrate utilization, and instead they specialized on using proteinaceous resources. Here we propose a new hypothesis to explain genomic reduction in this genus; we propose that trophic specialization increasing the metabolic efficiency for using one kind of substrate but reducing the substrate utilization spectrum could result in bacterial genomic reduction, which would be not uncommon in nature. This hypothesis was further tested in another free-living genus, *Kangiella*, which also shows dramatic genomic reduction. These findings highlight that trophic specialization is potentially an important path leading to genomic reduction in some marine free-living bacteria, which is distinct from the classical lineages like SAR11.

## Abstract (precis)

The streamlining hypothesis is usually used to explain the genomic reduction events in free-living bacteria like SAR11. However, we find that the genomic reduction phenomenon in the bacterial genus *Idiomarina* is different from that in SAR11. Therefore, we propose a new hypothesis to explain genomic reduction in this genus based on trophic specialization that could result in genomic reduction, which would be not uncommon in nature. Not only can the trophic specialization hypothesis explain the genomic reduction in the genus *Idiomarina*, but it also sheds new light on our understanding of the genomic reduction processes in other free-living bacterial lineages.

## INTRODUCTION

Bacterial genome size shows substantial variation. Known bacterial genome sizes can range from less than 0.5 megabase pairs (Mbp) to more than 10 Mbp (1, 2). Bacteria, especially the free-living ones, usually have a genome size of 3 to 6 Mbp. For example, for the well-studied *Gammaproteobacteria* (with more than 2,000 complete genomes), 77% have a genome size of 3 to 6 Mbp (data from the NCBI genome database [https://www.ncbi.nlm.nih.gov/genome/]). On the one hand, bacterial genome size can increase through horizontal gene transfer and gene duplication (3–6). Bacteria that have larger genomes are assumed to be versatile in their lifestyles and are presumably adapted to changing environments. On the other hand, bacterial genome size can decrease (genomic reduction) by losing genes, and bacteria that have small genomes seem to prefer stable habitats (2). Generally, there are two kinds of genomic reduction in bacteria. One kind is related to the host-associated bacteria such as parasites and symbionts of animals and plants. Another kind is related to the free-living bacteria, such as the cultured marine bacteria *Pelagibacter* and *Prochlorococcus* (2, 7) as well as several metagenome-derived uncultured bacteria (8–11).

The streamlining hypothesis is commonly used to explain the genomic reduction events in free-living bacteria, such as the marine bacteria *Pelagibacter* and *Prochlorococcus* living in stable, nutrient-poor environments (12–14). Genome streamlining is regarded as an adaptive genomic reduction process in which selection for metabolic efficiency could drive genome reduction. In stable, nutrient-poor environments, genomic reduction will have adaptive advantages, as losing sophisticated and costly regulatory and accessory machinery can reduce the metabolic burden of the cell, such as the reduced requirement for nitrogen and phosphorus, two elements that are rare in surface seawater. The common consequences of streamlined organisms include small cell and genome size, a low G+C content, extremely reduced intergenic spacers, loss of accessory machinery, and very low numbers of paralogs, pseudogenes, phage genes, and regulatory genes.

Research on the streamlining hypothesis has mainly focused on the well-studied marine free-living bacterial groups, such as *Prochlorococcus*, roseobacters, SAR11, and SAR86. Free-living bacteria that have reduced genomes are predicted to be common in nature (15, 16). Our current understanding of the relationship between bacterial genome size and their environmental adaptation relies on too few species. It is still unclear whether there are other types of genomic reduction in the free-living bacteria. Lifestyle could dramatically affect bacterial genomes, but the relationships between genome size and trophic lifestyles are complex, and generalizations are difficult to make (17, 18). Studying more natural genomic reduction cases will undoubtedly improve our understanding of the evolutionary forces that drive this process in free-living bacteria.

The free-living bacterial genus *Idiomarina* belongs to the order *Alteromonadales* of the well-studied class *Gammaproteobacteria* (19). At this point, more than 10 genomes of strains from this genus have been deposited into public databases. All the sequenced genomes had a size less than 3 Mbp, much smaller than that of the genomes from other genera of the order *Alteromonadales* (all with an average genome size larger than 4 Mbp). This shows that the genomes of the genus *Idiomarina* had suffered dramatic genomic reduction. By comparative genomic and physiological studies, we find that the genomic reduction pattern in this genus is distinct from the pattern in the classic lineages like SAR11. We here propose a new hypothesis that trophic specialization can result in genomic reduction in the free-living bacteria of this genus.

## RESULTS AND DISCUSSION

### Extensive genomic reduction in the genus *Idiomarina*.

**FIG 1:**
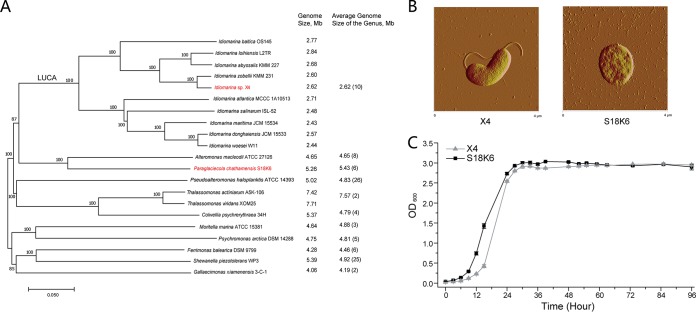
Phylogenetic and physiological analyses of the genus *Idiomarina*. (A) Phylogenetic tree and genomic size of species of the genus *Idiomarina* and related genera from the order *Alteromonadales*. The tree was constructed based on concatenated alignment of 685 single-copy orthologous proteins shared by all the genomes using the neighbor-joining method with 1,000 bootstrap replications. Genome size is shown in megabase pairs (Mb). The numbers in parentheses are the number of genomes used to calculate the average genome size of the genus. This tree was used to infer gene gain and loss events in the *Idiomarina* genus. LUCA indicates the position of the last universal common ancestor of the *Idiomarina* genus. The strains used for physiological analyses, namely, *Idiomarina* sp. strain X4 and Paraglaciecola chathamensis S18K6, are shown in red. All the species except strain X4 were formally identified. (B and C) Cells (B) and growth curves (C) of strains X4 and S18K6 cultured in 2216E medium.

Most species of the genus *Idiomarina* were isolated from deep-sea or high-salinity marine environments, and several genomes of this genus have been deposited into public databases (20–22). Ten bacteria of which nine were formally described from this genus that had defined isolation information are selected in this study. The bacterium *Idiomarina* sp. strain X4 (hereafter called X4) was isolated from the deep-sea sediment of the South China Sea at a water depth of 2,500 m by our laboratory, and we sequenced its complete genome. The average nucleotide identity and percentage of conserved protein values between strain X4 and its closest relative Idiomarina zobellii KMM 231 are 86.6% and 85.2%, respectively. On the basis of these two genomic classification standards (23, 24), X4 should belong to the genus *Idiomarina* but represent a new species. Because strain X4 is kept in our laboratory, this strain is also included in comparative genomic analyses and used for further physiological analyses. The size of the 10 *Idiomarina* genomes ranges from 2.44 to 2.84 Mbp with an average size of 2.62 Mbp, which is much smaller than that of the genomes from all the other studied genera of the order *Alteromonadales* (with average genome size of 4.19 to 7.57 Mbp) (Fig. 1A). This shows that the strains of the *Idiomarina* genus have suffered extensive genomic reduction, with 37% to 65% genome size reduction compared to other genera, in their evolutionary history. As no parasitic and commensal characteristics of this genus were observed, this offers a unique opportunity to investigate the genomic reduction process in free-living bacteria.

### The genomic reduction pattern in the *Idiomarina* genus is different from that of the classical SAR11 lineage.

The genomic reduction processes and characteristics were well studied in the SAR11 lineage like strain “*Candidatus* Pelagibacter ubique” HTCC1062 (hereafter called HTCC1062) (7). We then investigated whether *Idiomarina* species had genomic traits similar to those of HTCC1062. Besides HTCC1062, three *Idiomarina* species and three strains with larger genomes in the order *Alteromonadales* that were also isolated from the deep-sea environment are included for comparison (Table 1). The formally identified strain Paraglaciecola chathamensis S18K6 (hereafter called S18K6) that is a close relative to the *Idiomarina* genus is further used for physiological comparison (25, 26). The comparison results are somewhat contradictory. The *Idiomarina* species have some genomic characteristics similar to those of the SAR11 lineage, such as a high percentage of coding regions, no CRISPR site, and a low number of paralog clusters. However, the *Idiomarina* species have some genomic characteristics that are different from those of the SAR11 lineage and some genomic characteristics that are similar to those of relatives with larger genomes, such as high G+C content, high numbers of rRNA operons, regulatory sigma factors, and mismatch repair proteins, and high percentage of extracellular proteins, like peptidase. The genomic analyses show that the genomic traits of the genus *Idiomarina* are partially different from those of the SAR11 lineage.

**TABLE 1:** Comparison of the genomic characteristics of selected genomes

Genomic characteristic	“*Candidatus* Pelagibacter ubique” HTCC1062	Idiomarina loihiensis L2TR	*Idiomarina* sp. strain X4	Idiomarina abyssalis KMM 227	Colwellia psychrerythraea 34H	Paraglaciecola chathamensis S18K6	Shewanella piezotolerans WP3
Genome size (Mbp)	1.31	2.84	2.62	2.68	5.37	5.26	5.39
G+C content (%)	29.7	47.0	47.3	47.2	37.9	44.1	43.2
% coding	97.1	92.1	92.3	93.5	85.8	87.5	84.4
No. of rRNA operons	1	3	3	3	9	5	8
No. of CRISPR sites	0	0	0	0	0	2	1
No. of paralog clusters							
With 50% identity	9	30	22	20	145	99	101
With 80% identity	0	7	5	6	59	15	34
No. of sigma factors	4	14	10	12	19	18	8
% protein located outside the cell	0.5	1.1	1.1	1.0	1.2	1.3	1.5
Total no. of peptidase sequences (% of total proteins)	63 (4.6)	130 (5.0)	116 (4.7)	124 (5.0)	191 (3.9)	180 (3.8)	198 (4.0)
No. of peptidases located outside the cell	3	17	20	15	26	29	27
α-Amylase	0	0	0	0	4	5	5
β-Galactosidase	0	0	0	0	1	4	2
Alginate lyase	0	0	0	0	0	2	0
Total no. of carbohydrate-active enzyme sequences (% of total proteins)	27 (1.9)	26 (1.0)	25 (1.0)	26 (1.0)	83 (1.7)	109 (2.3)	63 (1.3)

In the SAR11 lineage, genomic reduction was usually accompanied by a low growth rate, small cell size, and loss of accessory structures of bacterial cells, such as flagella. However, the cells of identified *Idiomarina* species are usually 1 to 2 μm long and 0.5 to 1 μm wide (19, 27–29). The cell size of strain X4 (∼1 by 2 μm) falls in this range, which is comparable to the cell size of strain S18K6 (Fig. 1B) and much larger than the cell size of strain HTCC1062 (∼0.4 μm in diameter). In addition, a polar flagellum is observed for strain X4, which is consistent with the finding that all identified *Idiomarina* species had a polar flagellum and motility in liquid environment (19, 27–29). When grown in rich medium, strain X4 can reach a high OD_600_ value at a high speed comparable with that of strain S18K6 (Fig. 1C). The growth traits of strains X4 and S18K6 are obviously different from those of strain HTCC1062, which can reach undetectable OD_600_ values only in natural and laboratory environments. Therefore, the high growth rate and cell morphology of *Idiomarina* species are clearly different from those of the SAR11 lineage. Taken together, the genomic and physiological characteristics of the *Idiomarina* genus are different from those of the classical SAR11 lineage, and a new hypothesis is therefore needed to explain the different genomic reduction phenomenon in the genus *Idiomarina*.

Although adaptive selection has usually been used to explain streamlined genomic reduction, Luo et al. recently proposed that genetic drift could lead to ancient genomic reduction in some marine bacterial lineages, such as *Prochlorococcus*, roseobacters, and SAR86 (30). They found that the ratio of radical (*d*_R_) and conservative (*d*_C_) nonsynonymous nucleotide substitutions were elevated in the streamlined lineages compared to their relatives with larger genomes. We then tested the effect of genetic drift on genomic reduction in the genus *Idiomarina* using the methods described by Luo et al. (30). We find that compared to the control clade, the *d*_R_*/d*_C_ ratio is not significantly inflated in the *Idiomarina* genus (*P* > 0.05 [see Fig. S1 in the supplemental material]), suggesting that there is no excess of radical amino acid changes in the ancestral branch giving rise to the *Idiomarina* genus compared to its relatives with larger genomes. This result also shows that the mechanism and process of genomic reduction in the *Idiomarina* genus are different from those of other well-studied marine free-living bacterial lineages.

10.1128/mBio.02545-18.1 FIG S1 Analysis of conservative and radical nonsynonymous nucleotide substitutions in the *Idiomarina* genus and related strains with larger genomes. (A) Phylogenomic tree as in Fig. 1 with the target, control, and reference groups indicated. For calculation of the *d*_R_/*d*_C_ ratio, genomes in the target group and control group were each compared to those in the referece group, and then the *d*_R_/*d*_C_ ratio was computed and compared. (B and C) Box chart of the *d*_R_/*d*_C_ ratio calculated based on physicochemical classification of the 20 amino acids by charge (B) and polarity (C). Results were calculated using RCCalculater (reference 30) with uncorrected HON-NEW (uncorrected), GC corrections based on codon frequency (corrected with codon frequency), and GC corrections based on amino acid compostion (corrected with amino acid composition) methods. The *P* values are from a paired *t* test. Download FIG S1, TIF file, 1.5 MB. Copyright © 2019 Qin et al. 2019 Qin et al. This content is distributed under the terms of the Creative Commons Attribution 4.0 International license.

### Reconstruction of the genomic reduction process in the genus *Idiomarina*.

**FIG 2:**
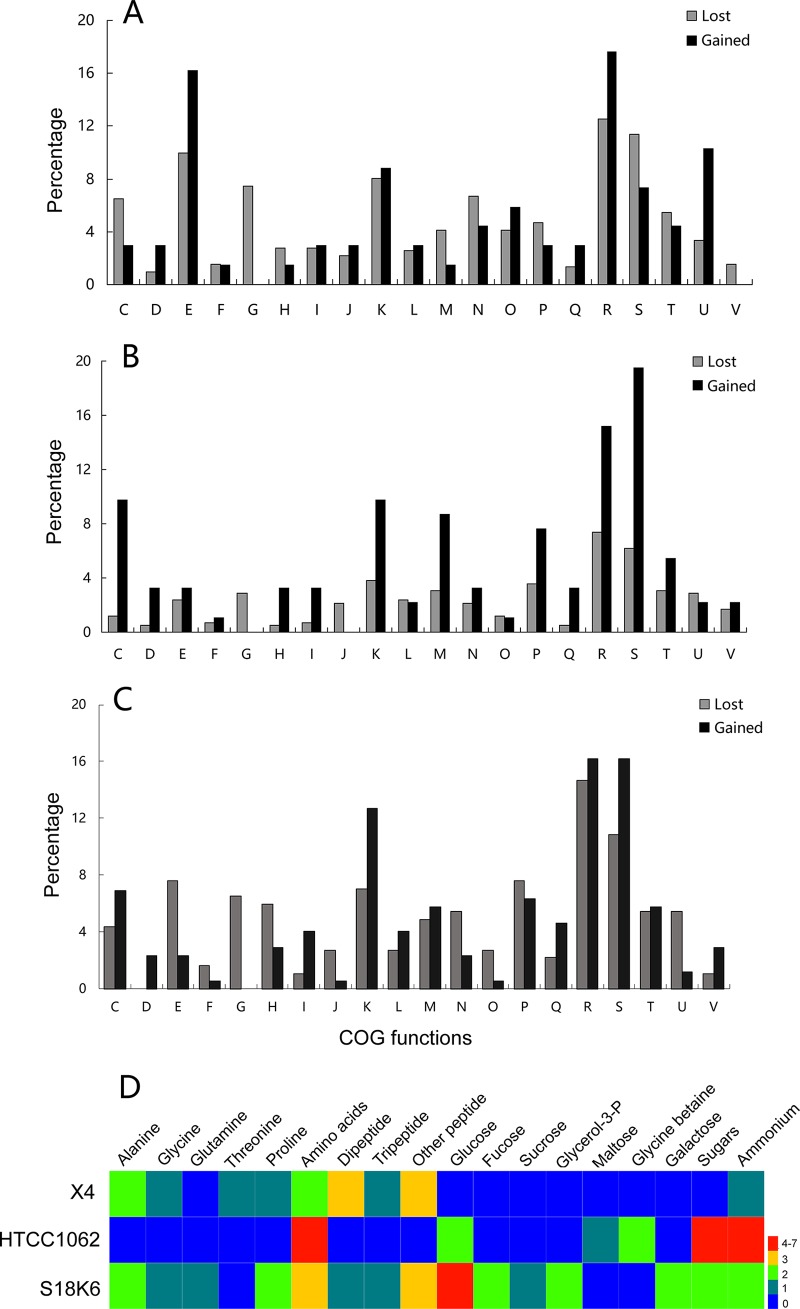
Comparison of the identified COG functions of lost and gained genes (A to C) and heatmap of identified transporters in three strains (D). (A) The identified COG functions of lost and gained genes in the last universal common ancestor (LUCA) of the genus *Idiomarina*. (B and C) The identified COG functions of lost and gained genes in the evolutionary path from the LUCA to the X4 (B) and *I. loihiensis* L2TR (C) terminus. COG functional categories are shown on the *x* axis and are as follows: C, energy production and conversion; E, amino acid transport and metabolism; F, nucleotide transport and metabolism; G, carbohydrate transport and metabolism; H, coenzyme transport and metabolism; I, lipid transport and metabolism; J, translation, ribosomal structure functions, and biogenesis; K, transcription; L, replication, recombination, and repair; M, cell wall/membrane/envelope biogenesis; O, posttranslational modification, protein turnover, or chaperone functions; P, inorganic ion transport and metabolism; Q, secondary metabolite functions; R, general function prediction only; S. unknown function; T, signal transduction mechanism functions; U, intracellular trafficking, secretion, and vesicular transport; V, defense mechanism functions.

The gene gain and loss events were reconstructed to investigate the genomic reduction process in *Idiomarina*. The last universal common ancestor (LUCA) (containing 2,164 gene clusters) of the genus *Idiomarina* had gained 135 gene clusters and lost 685 gene clusters, showing that the LUCA of this genus already had suffered great genomic reduction. Approximately 45% and 64% of the gained and lost gene clusters, respectively, can be assigned a COG function. The COG functional analyses show that high proportions of the genes lost are related to energy production and conversion (COG category C), carbohydrate transport and metabolism (G), cell wall/membrane/envelope biogenesis (M), and defense mechanisms (V), while the genes gained are enriched in functions of amino acid transport and metabolism (E), general predicted function (R), and intracellular trafficking, secretion, and vesicular transport (U) (Fig. 2A). In particular, note that 38 gene clusters related to carbohydrate transport and metabolism (category G) were lost, while no genes related to this function were gained.

Gene gain and loss events are further analyzed in the evolutionary path from the LUCA to the Idiomarina loihiensis L2TR and X4 terminus. A striking feature of this evolutionary path is that both strains continued to lose carbohydrate metabolism genes in the genomic reduction process (Fig. 2B and C). Consistent with this, there are only 25 or 26 carbohydrate-active enzyme sequences in the *Idiomarina* genomes compared, even less than that in strain HTCC1062 and much less than that in related strains with larger genomes (Table 1). The *Idiomarina* genomes also do not harbor genes encoding high-molecular-weight sugar polymer-degrading enzymes that are encoded in other strains (Table 1). Meanwhile, both strains consistently gained genes related to amino acid transport and metabolism (Fig. 2B and C). For example, the percentage of the peptidases that can degrade proteinaceous substrates and initiate amino acid utilization is extremely high in the *Idiomarina* genomes (Table 1). The *Idiomarina* genomes encode 15 to 20 peptidases (belonging to metallo, serine, and threonine families) predicted outside the cell membrane. The activity of peptidases can produce amino acids or peptides. Accordingly, genes encoding the transporters to absorb small peptides as well as some amino acids are found in the X4 genome, while the transporters responsible for absorbing small sugars are not found (Fig. 2D). This shows that strain X4 would have strong protein degradation ability and that it mainly acquires carbon and energy from proteinaceous resources, as previously reported for I. loihiensis L2TR, which was also isolated from the deep sea (31). *I. loihiensis* L2TR lost many carbohydrate metabolism genes, such as the genes encoding transaldolase, glucose-6-phosphate dehydrogenase, and 2-keto-3-deoxy-6-phosphogluconate aldolase (31), which are also absent in the X4 genome. Strain X4 lost a total of 36 identified transcriptional regulator genes, including one clearly annotated as relating to sugar metabolism (COG1349). This implies that when carbohydrate utilization genes are lost, related regulatory genes are also lost.

The reconstruction of the genomic reduction process in the *Idiomarina* genus showed that many more genes were lost than gained. Generally, the lost and gained genes can be assigned to all COG function categories except that carbohydrate transport and metabolism genes were consistently lost (Fig. 2). Meanwhile, the *Idiomarina* genus gained many genes related to utilization of proteinaceous substrates.

### Trophic specialization of the genus *Idiomarina*.

**FIG 3:**
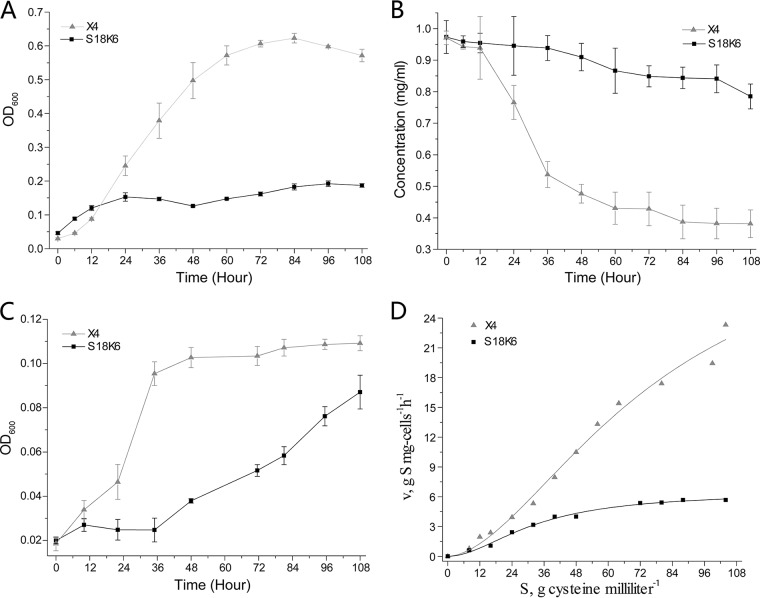
Comparison of the proteinaceous substrate utilization abilities of strains X4 and S18K6. (A) Growth curves of both strains cultured in 0.1% casein medium. (B) Concentrations of casein in the medium when both strains were cultured in 0.1% casein medium. (C) Growth curves of both strains cultured in 0.002% casein medium. (D) Cysteine absorption efficiencies for both strains.

Genome evolutionary reconstruction indicated that members of the genus *Idiomarina*, including strain X4, lost many genes related to sugar utilization and had to rely on, and even developed, the ability to use exogenous proteinaceous substrates as carbon and energy resources. This genomic prediction was further tested experimentally in strain X4. The substrate utilization profiles showed that glucose was the only sugar that supported the growth of strain X4 and that its ability to use a variety of other sugars as carbon and energy sources was limited (Table 2). The identified species of this genus also showed limited sugar utilization ability (19, 27–29), which is in consistent with the genomic analyses that all species of the genus had lost many sugar utilization genes. Meanwhile, strain X4 has evolved a strong ability to use proteinaceous substrates. In 0.1% casein medium, strain X4 can grow more quickly and reach a higher cell density than strain S18K6 (Fig. 3A), while these two strains had comparable growth rates in rich medium (Fig. 1C). Consistent with its high growth rate, strain X4 can consume casein more quickly than strain S18K6 can (Fig. 3B). In addition, strain X4 responded and grew much more quickly than S18K6 in 0.002% (∼10 mg carbon per liter) casein medium (Fig. 3C), showing that X4 can adapt to utilize low concentrations of proteinaceous substrates. Degradation of high-molecular-weight proteinaceous substrates can produce small peptides and amino acids, and accordingly, strain X4 carries genes that encode transporters for peptides and amino acids. The amino acid cysteine absorption rates of these two strains were further tested, and the results showed that X4 cells had a higher cysteine absorption rate than S18K6 cells, especially when the cysteine concentration was high (Fig. 3D). The genomic and experimental analyses indicate that strain X4 is specialized in using proteinaceous substrates as nutrition and energy resources, which was also reported for the deep-sea bacterium *I. loihiensis* L2TR (31). This implies that trophic specialization in using proteinaceous substrates in *Idiomarina* species may be a strategy to adapt to the deep-sea environment, as it has been reported that high-molecular-weight (HMW) dissolved organic nitrogen in the deep sea is mainly of proteinaceous origin and specific proteases were required to degrade these HMW proteinaceous substrates (32, 33).

**TABLE 2:** Substrate utilization profiles of *Idiomarina* sp. strain X4 and Paraglaciecola chathamensis S18K6

Substrate	*Idiomarina* sp. strain X4		P. chathamensis S18K6	
	Expected from genomic analysis	Experimental results^*a*^	Expected from genomic analysis	Experimental results
Glucose	+	+	+	+
Mannose	−	−	+	+
Fructose	−	−	+	+
Sucrose	−	−	+	+
Glycerol	−	−	+	+
Maltose	−	−	+	+
CMC-Na^*b*^	−	−	+	−
Galactose	−	−	+	+
Starch	−	−	+	+
Xylan	−	−	+	+
Alginate-Na	−	−	+	−
Casein	+	+	+	+
Gelatin	+	+	+	+
Elastin	+	−	+	−

a Symbols: +, the OD_600_ of cultures was >0.1 after 6 days’ growth; −, the OD_600_ was <0.05 after 6 days’ growth.

b CMC-Na, carboxymethylcellulose-Na.

### Correlation between genomic reduction and trophic specialization.

The genomic and physiological analyses indicate that genomic reduction is related to trophic specialization in the genus *Idiomarina*. However, the correlation between genomic reduction and trophic specialization is hard to elucidate. The strain may have first lost the sugar utilization genes, then it had to rely on proteinaceous resources, and trophic specialization developed further. It also may be that the strain had first developed and relied on the ability to use proteinaceous resources, and then the sugar utilization genes were less used under natural selection and were finally lost from the genome. There is a third possibility that genomic reduction and trophic specialization are the result of other selective pressure. We propose that trophic specialization evolved first. The lost sugar utilization genes such as glucose-6-phosphate dehydrogenase and 2-keto-3-deoxy-6-phosphogluconate aldolase are usually essential genes in other bacteria (34, 35). The ancestor of the genus *Idiomarina* would not survive if it lost these genes but did not develop protein utilization ability. If the ancestor developed the protein utilization ability first, then the sugar utilization genes would not be under stringent selection and could be lost from the genome. Most strains of the genus *Idiomarina* were isolated from high-salinity or deep-sea environments, both of which are extreme conditions. In order to survive in these extreme environments, bacteria have to develop special adaptation abilities, of which obtaining nutrients is the basic and most important one. We then propose a hypothesis that adaptation to the special environment with trophic specialization narrowing the food spectrum could result in genomic reduction.

This would not be uncommon in nature. Mutations that increase the activity of one process are likely to affect other processes, which can promote the emergence of mutants that specialize in metabolizing certain substrates (36, 37). In cases where the ancestor had multiple abilities for substrate utilization, an adaptive trait could have evolved in response to a special environment through specialization for using one kind of substrate with higher efficiency. If the remaining genes for utilization of other substrates become nonessential in this special environment, these genes would no longer be under strong purifying selection, allowing for the accumulation of deleterious mutations and eventually leading to permanent deletion of these genes and related regulatory genes, thus resulting in genome size reduction. Interestingly, a recent study showed that a *Polaribacter* isolate preferring to feed on proteins also had a reduced genome size compared to its close relative that had more polysaccharide utilization genes and a broader carbohydrate utilization spectrum (38).

### Testing the trophic specialization hypothesis in other bacterial genomes.

**FIG 4:**
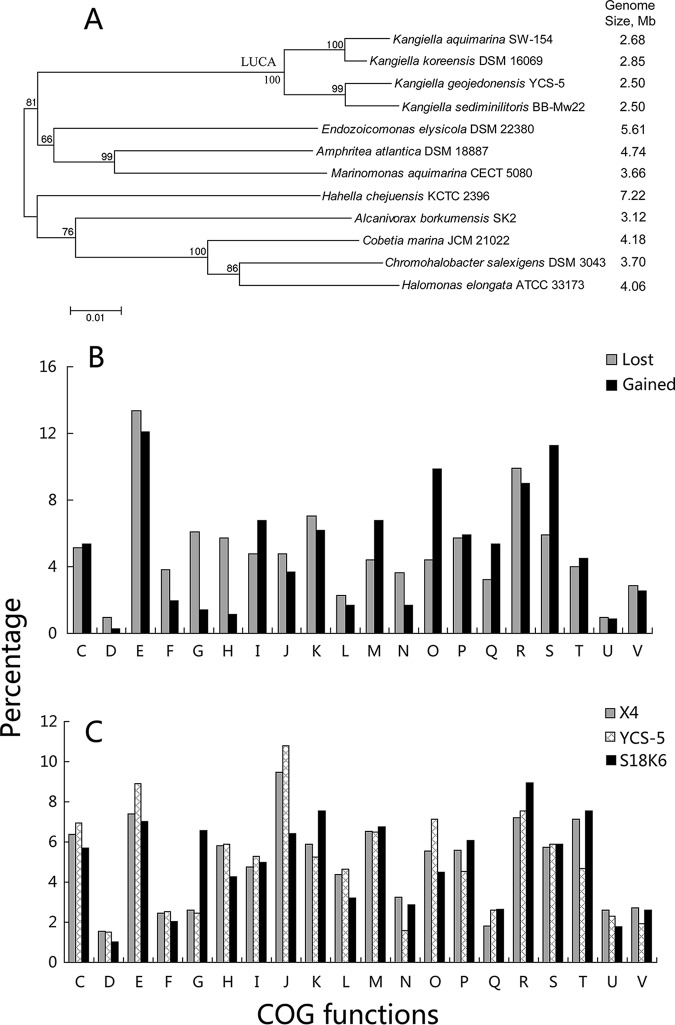
Genomic reduction analyses of the genus *Kangiella.* (A) Phylogenetic tree and genome size (in megabase pairs [Mb]) of the genus *Kangiella* and related genera, showing the dramatic genomic reduction in the genus *Kangiella*. (B) Comparison of the identified COG functions of lost and gained genes in the LUCA of the genus *Kangiella*. (C) Comparison of the identified COG functions of all the genes of strains X4, Kangiella geojedonensis YCS-5, and S18K6. COG functional categories are given in the legend to Fig. 2.

We searched the bacterial genomes belonging to the well-studied *Gammaproteobacteria* (>2,000 complete genomes) with genome sizes of 2 to 3 Mbp, and 131 genomes, including 2 *Idiomarina* genomes, were found. Of these 131 bacterial genomes, ∼90% are parasites or pathogens of animals or plants and belong to genera such as *Actinobacillus*, *Coxiella*, *Haemophilus*, *Pasteurella,* and *Xylella*. These parasites or pathogens can be considered another type of trophic specialization (see discussion below). One genus that suffered dramatic genomic reduction and was not a parasite or a pathogen is *Kangiella* (four genomes) (Fig. 4A) (39, 40). Therefore, the genomic reduction processes of this genus was reconstructed. The LUCA of the genus *Kangiella* had gained 538 gene clusters, while it had lost 596 gene clusters, showing that the LUCA of this genus suffered some extent of genomic reconstruction. A striking feature is that the LUCA also lost a high proportion of genes related to carbohydrate transport and metabolism (G), including an ABC-type sugar transport system, and had gained a high proportion of genes related to amino acid transport and metabolism (E), including 15 peptidases (Fig. 4B). This implies that the genus *Kangiella*, like the genus *Idiomarina*, had lost some carbohydrate utilization ability and developed the ability to use proteinaceous substrates. Accordingly, the proportion of carbohydrate utilization genes (G) in Kangiella geojedonensis YCS-5 is comparable to that in strain X4 and much lower than that in strain S18K6 (2.5% versus 6.6%), while the differences of other functions are not as large (Fig. 4C). The physiological traits showed that K. geojedonensis YCS-5 could hydrolyze casein, gelatin, and tyrosine, but not starch (41). The other three strains Kangiella koreensis DSM 16069, K. aquimarina SW-154, and K. sediminilitoris BB-Mw22 also could hydrolyze casein and tyrosine, but not starch (40, 42). The genomic and phenotypic characteristics also indicate that genome reduction in the genus *Kangiella* is accompanied by a certain extent of trophic specialization.

### Conclusion.

This study indeed illustrates there are genomic reduction patterns and processes different from those in the classical free-living lineages such as SAR11. We propose that selection for trophic specialization in certain environments would be an important path leading to genomic reduction in the genus *Idiomarina*. Not only can the trophic specialization hypothesis explain genomic reduction in the genus *Idiomarina*, but it also sheds new light on the understanding of the genomic reduction processes in other marine free-living bacteria.

## MATERIALS AND METHODS

### Bacterial strains and phenotypic characteristics.

*Idiomarina* sp. strain X4 was isolated by and maintained in our laboratory. The Paraglaciecola chathamensis S18K6 strain was purchased from the culture collection center as described previously (43). Both strains were routinely cultured using 2216E broth medium: 5 g peptone, 1 g yeast extract, 1 liter artificial seawater (containing 35 g sea salt [Sigma, USA]), pH 7.5. To investigate the ability to use proteinaceous substrates, 0.1% or 0.002% casein media (1 g or 0.02 g casein, 1× vitamin mix, 1 liter artificial seawater, pH 7.5) was used to culture both strains. To test the substrate utilization profiles, the media containing different substrates (substrates tested are shown in Table 2), 1× vitamin mix, 1 liter artificial seawater, 0.01 g NH_4_NO_3_ for sugar substrate tests, and pH 7.5, were used. The growth of both strains cultured in proteinaceous and sugar-containing media at 18°C with a shaking speed of 180 rpm was quantified by measuring the optical density of the cultures at 600 nm (OD_600_). Cysteine absorption rates were measured by the method of Button (44). The cysteine concentration was assayed by an L-8900 amino acid analyzer (Hitachi, Japan). Micrographs of the strains were obtained in air in ScanAsyst mode by atomic force microscopy (AFM) using a Multimode Nanoscope VIII AFM (Bruker AXS, Germany) with probe NSC11 (MikroMasch, USA).

### Genome sequencing and bioinformatic analyses.

The complete genome of *Idiomarina* sp. strain X4 was sequenced by using a combination of second (Illumina HiSeq2000) and third (PacBio RS II) sequencing platforms. The genome was annotated using the RAST annotation pipeline (45). Metabolic pathways were determined using the online KEGG mapping tool (46). Carbohydrate-active enzyme sequences and modules were analyzed using the CAZy database (47). The COG function category was analyzed by searching proteins against the updated COG database using the blastp program. The protein subcellular localization was predicted using PSORTb 3.0 (48). Transporters were identified by searching proteins against the transporter classification database (TCDB) (49). The final results of COG function, protein subcellular localization, and transporter identification were put together by custom-made Perl scripts. The orthologous clusters were grouped using OrthoMCL (50). Amino acid sequences of single-copy orthologous proteins shared by all the genomes were aligned using MUSCLE (51). The alignments were concatenated, and the phylogenetic tree was constructed using MEGA 7.0 with the neighbor-joining method (52). Count software was used to infer the gene gain and loss events in the *Idiomarina* and *Kangiella* genera with the posterior probability model (53).

### Accession number(s).

The complete genome sequence of *Idiomarina* sp. strain X4 was deposited in GenBank under accession no. CP025000.

## References

[1] Moran NA 2002 Microbial minimalism: genome reduction in bacterial pathogens. Cell 108:583–586. doi:10.1016/S0092-8674(02)00665-7. 11893328

[2] Batut B, Knibbe C, Marais G, Daubin V 2014 Reductive genome evolution at both ends of the bacterial population size spectrum. Nat Rev Microbiol 12:841–850. doi:10.1038/nrmicro3331. 25220308

[3] de la Cruz F, Davies J 2000 Horizontal gene transfer and the origin of species: lessons from bacteria. Trends Microbiol 8:128–133. doi:10.1016/S0966-842X(00)01703-0. 10707066

[4] Abby S, Daubin V 2007 Comparative genomics and the evolution of prokaryotes. Trends Microbiol 15:135–141. doi:10.1016/j.tim.2007.01.007. 17289390

[5] Zhaxybayeva O, Doolittle WF 2011 Lateral gene transfer. Curr Biol 21:R242–R246. doi:10.1016/j.cub.2011.01.045. 21481756

[6] Sela I, Wolf YI, Koonin EV 2016 Theory of prokaryotic genome evolution. Proc Natl Acad Sci U S A 113:11399–11407. doi:10.1073/pnas.1614083113. 27702904 PMC5068321

[7] Giovannoni SJ, Tripp HJ, Givan S, Podar M, Vergin KL, Baptista D, Bibbs L, Eads J, Richardson TH, Noordewier M, Rappe MS, Short JM, Carrington JC, Mathur EJ 2005 Genome streamlining in a cosmopolitan oceanic bacterium. Science 309:1242–1245. doi:10.1126/science.1114057. 16109880

[8] Albertsen M, Hugenholtz P, Skarshewski A, Nielsen KL, Tyson GW, Nielsen PH 2013 Genome sequences of rare, uncultured bacteria obtained by differential coverage binning of multiple metagenomes. Nat Biotechnol 31:533–538. doi:10.1038/nbt.2579. 23707974

[9] Ghai R, Mizuno CM, Picazo A, Camacho A, Rodriguez-Valera F 2013 Metagenomics uncovers a new group of low GC and ultra-small marine Actinobacteria. Sci Rep 3:2471. doi:10.1038/srep02471. 23959135 PMC3747508

[10] Luo H, Loytynoja A, Moran MA 2012 Genome content of uncultivated marine Roseobacters in the surface ocean. Environ Microbiol 14:41–51. doi:10.1111/j.1462-2920.2011.02528.x. 21854517

[11] Luo H, Swan BK, Stepanauskas R, Hughes AL, Moran MA 2014 Evolutionary analysis of a streamlined lineage of surface ocean Roseobacters. ISME J 8:1428–1439. doi:10.1038/ismej.2013.248. 24451207 PMC4069390

[12] Giovannoni SJ, Cameron Thrash J, Temperton B 2014 Implications of streamlining theory for microbial ecology. ISME J 8:1553–1565. doi:10.1038/ismej.2014.60. 24739623 PMC4817614

[13] Martínez-Cano DJ, Reyes-Prieto M, Martínez-Romero E, Partida-Martínez LP, Latorre A, Moya A, Delaye L 2014 Evolution of small prokaryotic genomes. Front Microbiol 5:742. doi:10.3389/fmicb.2014.00742. 25610432 PMC4285135

[14] Wernegreen JJ 2015 Endosymbiont evolution: predictions from theory and surprises from genomes. Ann N Y Acad Sci 1360:16–35. doi:10.1111/nyas.12740. 25866055 PMC4600008

[15] Swan BK, Tupper B, Sczyrba A, Lauro FM, Martinez-Garcia M, Gonzalez JM, Luo H, Wright JJ, Landry ZC, Hanson NW, Thompson BP, Poulton NJ, Schwientek P, Acinas SG, Giovannoni SJ, Moran MA, Hallam SJ, Cavicchioli R, Woyke T, Stepanauskas R 2013 Prevalent genome streamlining and latitudinal divergence of planktonic bacteria in the surface ocean. Proc Natl Acad Sci U S A 110:11463–11468. doi:10.1073/pnas.1304246110. 23801761 PMC3710821

[16] Wolf YI, Koonin EV 2013 Genome reduction as the dominant mode of evolution. Bioessays 35:829–837. doi:10.1002/bies.201300037. 23801028 PMC3840695

[17] Karcagi I, Draskovits G, Umenhoffer K, Fekete G, Kovacs K, Mehi O, Baliko G, Szappanos B, Gyorfy Z, Feher T, Bogos B, Blattner FR, Pal C, Posfai G, Papp B 2016 Indispensability of horizontally transferred genes and its impact on bacterial genome streamlining. Mol Biol Evol 33:1257–1269. doi:10.1093/molbev/msw009. 26769030 PMC5854090

[18] Grote J, Thrash JC, Huggett MJ, Landry ZC, Carini P, Giovannoni SJ, Rappe MS 2012 Streamlining and core genome conservation among highly divergent members of the SAR11 clade. mBio 3:e00252-16. doi:10.1128/mBio.00252-12. 22991429 PMC3448164

[19] Zachariah S, Das SK 2017 *Idiomarina andamanensis* sp. nov., an alkalitolerant bacterium isolated from Andaman Sea water. Antonie Van Leeuwenhoek 110:1581–1592. doi:10.1007/s10482-017-0908-5. 28730371

[20] Mithoefer S, Rheaume BA, MacLea KS 2015 Draft whole-genome sequence of the marine bacterium *Idiomarina zobellii* KMM 231^T^. Genome Announc 3:e01257-15. doi:10.1128/genomeA.01257-15. 26514764 PMC4626610

[21] Gupta HK, Singh A, Sharma R 2011 Genome sequence of *Idiomarina* sp. strain A28L, isolated from Pangong Lake, India. J Bacteriol 193:5875–5876. doi:10.1128/JB.05648-11. 21742887 PMC3187211

[22] Lepcha RT, Poddar A, Whitman WB, Das SK 2016 Draft genome sequence of *Idiomarina woesei* strain W11^T^ (DSM 27808T) isolated from the Andaman Sea. Genome Announc 4:e00831-16. doi:10.1128/genomeA.00831-16. 27516518 PMC4982297

[23] Richter M, Rosselló-Móra R 2009 Shifting the genomic gold standard for the prokaryotic species definition. Proc Natl Acad Sci U S A 106:19126–19131. doi:10.1073/pnas.0906412106. 19855009 PMC2776425

[24] Qin QL, Xie BB, Zhang XY, Chen XL, Zhou BC, Zhou J, Oren A, Zhang YZ 2014 A proposed genus boundary for the prokaryotes based on genomic insights. J Bacteriol 196:2210–2215. doi:10.1128/JB.01688-14. 24706738 PMC4054180

[25] Shivaji S, Reddy GS 2014 Phylogenetic analyses of the genus *Glaciecola*: emended description of the genus *Glaciecola*, transfer of *Glaciecola mesophila*, *G. agarilytica*, *G. aquimarina*, *G. arctica, G. chathamensis, G. polaris* and *G. psychrophila* to the genus *Paraglaciecola* gen. nov. as *Paraglaciecola mesophila* comb. nov., *P. agarilytica* comb. nov., *P. aquimarina* comb. nov., *P. arctica* comb. nov., *P. chathamensis* comb. nov., *P. polaris* comb. nov. and *P. psychrophila* comb. nov., and description of *Paraglaciecola oceanifecundans* sp. nov., isolated from the Southern Ocean. Int J Syst Evol Microbiol 64:3264–3275. doi:10.1099/ijs.0.065409-0. 24981324

[26] Matsuyama H, Hirabayashi T, Kasahara H, Minami H, Hoshino T, Yumoto I 2006 *Glaciecola chathamensis* sp. nov., a novel marine polysaccharide-producing bacterium. Int J Syst Evol Microbiol 56:2883–2886. doi:10.1099/ijs.0.64413-0. 17158992

[27] Ivanova EP, Romanenko LA, Chun J, Matte MH, Matte GR, Mikhailov VV, Svetashev VI, Huq A, Maugel T, Colwell RR 2000 *Idiomarina* gen. nov., comprising novel indigenous deep-sea bacteria from the Pacific Ocean, including descriptions of two species, *Idiomarina abyssalis* sp. nov. and *Idiomarina zobellii* sp. nov. Int J Syst Evol Microbiol 50:901–907. doi:10.1099/00207713-50-2-901. 10758902

[28] Donachie SP, Hou S, Gregory TS, Malahoff A, Alam M 2003 *Idiomarina loihiensis* sp. nov., a halophilic gamma-Proteobacterium from the Lo'ihi submarine volcano, Hawai'i. Int J Syst Evol Microbiol 53:1873–1879. doi:10.1099/ijs.0.02701-0. 14657116

[29] Choi DH, Cho BC 2005 *Idiomarina seosinensis* sp. nov., isolated from hypersaline water of a solar saltern in Korea. Int J Syst Evol Microbiol 55:379–383. doi:10.1099/ijs.0.63365-0. 15653904

[30] Luo H, Huang Y, Stepanauskas R, Tang J 2017 Excess of non-conservative amino acid changes in marine bacterioplankton lineages with reduced genomes. Nat Microbiol 2:17091. doi:10.1038/nmicrobiol.2017.91. 28604700

[31] Hou S, Saw JH, Lee KS, Freitas TA, Belisle C, Kawarabayasi Y, Donachie SP, Pikina A, Galperin MY, Koonin EV, Makarova KS, Omelchenko MV, Sorokin A, Wolf YI, Li QX, Keum YS, Campbell S, Denery J, Aizawa S, Shibata S, Malahoff A, Alam M 2004 Genome sequence of the deep-sea gamma-proteobacterium *Idiomarina loihiensis* reveals amino acid fermentation as a source of carbon and energy. Proc Natl Acad Sci U S A 101:18036–18041. doi:10.1073/pnas.0407638102. 15596722 PMC539801

[32] Aluwihare LI, Repeta DJ, Pantoja S, Johnson CG 2005 Two chemically distinct pools of organic nitrogen accumulate in the ocean. Science 308:1007–1010. doi:10.1126/science.1108925. 15890880

[33] Ran LY, Su HN, Zhou MY, Wang L, Chen XL, Xie BB, Song XY, Shi M, Qin QL, Pang X, Zhou BC, Zhang YZ, Zhang XY 2014 Characterization of a novel subtilisin-like protease myroicolsin from deep sea bacterium *Myroides profundi* D25 and molecular insight into its collagenolytic mechanism. J Biol Chem 289:6041–6053. doi:10.1074/jbc.M113.513861. 24429289 PMC3937671

[34] Tian WN, Braunstein LD, Pang J, Stuhlmeier KM, Xi QC, Tian X, Stanton RC 1998 Importance of glucose-6-phosphate dehydrogenase activity for cell growth. J Biol Chem 273:10609–10617. doi:10.1074/jbc.273.17.10609. 9553122

[35] Fuhrman LK, Wanken A, Nickerson KW, Conway T 1998 Rapid accumulation of intracellular 2-keto-3-deoxy-6-phosphogluconate in an Entner-Doudoroff aldolase mutant results in bacteriostasis. FEMS Microbiol Lett 159:261–266. doi:10.1111/j.1574-6968.1998.tb12870.x. 9503620

[36] Luo H, Moran MA 2015 How do divergent ecological strategies emerge among marine bacterioplankton lineages? Trends Microbiol 23:577–584. doi:10.1016/j.tim.2015.05.004. 26051014

[37] Barve A, Wagner A 2013 A latent capacity for evolutionary innovation through exaptation in metabolic systems. Nature 500:203–206. doi:10.1038/nature12301. 23851393

[38] Xing P, Hahnke RL, Unfried F, Markert S, Huang S, Barbeyron T, Harder J, Becher D, Schweder T, Glockner FO, Amann RI, Teeling H 2015 Niches of two polysaccharide-degrading *Polaribacter* isolates from the North Sea during a spring diatom bloom. ISME J 9:1410–1422. doi:10.1038/ismej.2014.225. 25478683 PMC4438327

[39] Choe H, Kim S, Oh J, Nasir A, Kim BK, Kim KM 2015 Complete genome of *Kangiella geojedonensis* KCTC 23420^T^, putative evidence for recent genome reduction in marine environments. Mar Genomics 24:215–217. doi:10.1016/j.margen.2015.05.015. 26044616

[40] Yoon JH, Oh TK, Park YH 2004 *Kangiella koreensis* gen. nov., sp. nov. and *Kangiella aquimarina* sp. nov., isolated from a tidal flat of the Yellow Sea in Korea. Int J Syst Evol Microbiol 54:1829–1835. doi:10.1099/ijs.0.63156-0. 15388751

[41] Yoon JH, Kang SJ, Lee SY, Lee JS, Oh TK 2012 *Kangiella geojedonensis* sp. nov., isolated from seawater. Int J Syst Evol Microbiol 62:511–514. doi:10.1099/ijs.0.029314-0. 21478391

[42] Lee SY, Park S, Oh TK, Yoon JH 2013 *Kangiella sediminilitoris* sp. nov., isolated from a tidal flat sediment. Int J Syst Evol Microbiol 63:1001–1006. doi:10.1099/ijs.0.040691-0. 22685109

[43] Qin QL, Xie BB, Yu Y, Shu YL, Rong JC, Zhang YJ, Zhao DL, Chen XL, Zhang XY, Chen B, Zhou BC, Zhang YZ 2014 Comparative genomics of the marine bacterial genus Glaciecola reveals the high degree of genomic diversity and genomic characteristic for cold adaptation. Environ Microbiol 16:1642–1653. doi:10.1111/1462-2920.12318. 25009843

[44] Button DK 1998 Nutrient uptake by microorganisms according to kinetic parameters from theory as related to cytoarchitecture. Microbiol Mol Biol Rev 62:636–645. 9729603 10.1128/mmbr.62.3.636-645.1998 PMC98928

[45] Overbeek R, Olson R, Pusch GD, Olsen GJ, Davis JJ, Disz T, Edwards RA, Gerdes S, Parrello B, Shukla M, Vonstein V, Wattam AR, Xia F, Stevens R 2014 The SEED and the Rapid Annotation of microbial genomes using Subsystems Technology (RAST). Nucleic Acids Res 42:D206–D214. doi:10.1093/nar/gkt1226. 24293654 PMC3965101

[46] Du J, Yuan Z, Ma Z, Song J, Xie X, Chen Y 2014 KEGG-PATH: Kyoto encyclopedia of genes and genomes-based pathway analysis using a path analysis model. Mol Biosyst 10:2441–2447. doi:10.1039/c4mb00287c. 24994036

[47] Lombard V, Golaconda Ramulu H, Drula E, Coutinho PM, Henrissat B 2014 The carbohydrate-active enzymes database (CAZy) in 2013. Nucleic Acids Res 42:D490–D495. doi:10.1093/nar/gkt1178. 24270786 PMC3965031

[48] Yu NY, Wagner JR, Laird MR, Melli G, Rey S, Lo R, Dao P, Sahinalp SC, Ester M, Foster LJ, Brinkman FS 2010 PSORTb 3.0: improved protein subcellular localization prediction with refined localization subcategories and predictive capabilities for all prokaryotes. Bioinformatics 26:1608–1615. doi:10.1093/bioinformatics/btq249. 20472543 PMC2887053

[49] Saier MH Jr, Reddy VS, Tsu BV, Ahmed MS, Li C, Moreno-Hagelsieb G 2016 The Transporter Classification Database (TCDB): recent advances. Nucleic Acids Res 44:D372–D379. doi:10.1093/nar/gkv1103. 26546518 PMC4702804

[50] Fischer S, Brunk BP, Chen F, Gao X, Harb OS, Iodice JB, Shanmugam D, Roos DS, Stoeckert CJ Jr. 2011 Using OrthoMCL to assign proteins to OrthoMCL-DB groups or to cluster proteomes into new ortholog groups. Curr Protoc Bioinformatics Chapter 6:Unit 6.12.1-19. doi:10.1002/0471250953.bi0612s35. PMC3196566 21901743

[51] Edgar RC 2004 MUSCLE: multiple sequence alignment with high accuracy and high throughput. Nucleic Acids Res 32:1792–1797. doi:10.1093/nar/gkh340. 15034147 PMC390337

[52] Caspermeyer J 2016 MEGA evolutionary software re-engineered to handle today’s big data demands. Mol Biol Evol 33:1887. doi:10.1093/molbev/msw074. 27189553

[53] Csuros M 2010 Count: evolutionary analysis of phylogenetic profiles with parsimony and likelihood. Bioinformatics 26:1910–1912. doi:10.1093/bioinformatics/btq315. 20551134

