# Supplementary figures and images for: Trophic Specialization Results in Genomic Reduction in Free-Living Marine Idiomarina Bacteria
Source: mBio. 2019 Jan 15;10(1):e02545-18. doi: 10.1128/mBio.02545-18 (PMC6336423; doi:10.1128/mBio.02545-18)

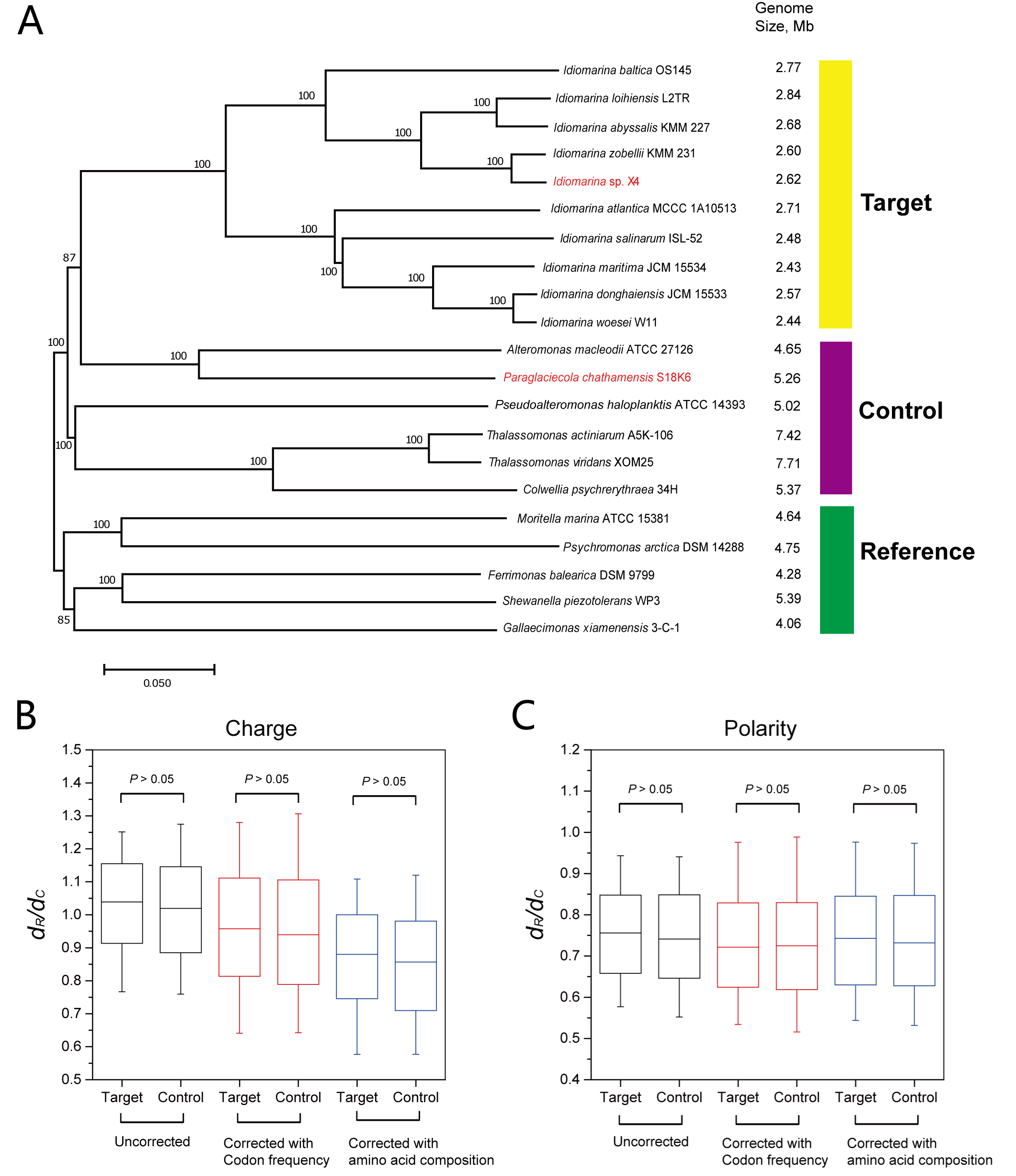

Supplement: FIG S1 [file mBio.02545-18-sf001.tif]
